# Supplementary material for: Transcriptomic Insights into Mechanisms of Early Seed Maturation in the Garden Pea (Pisum sativum L.)
Source: Cells. 2020 Mar 23;9(3):779. doi: 10.3390/cells9030779 (PMC7140803; doi:10.3390/cells9030779)

Sprint−2,20(4)

Global Summary

%DE = 0.23

genes with fdr < 0.2 = 471 (220 + / 251 −)

genes with fdr < 0.1 = 435 (206 + / 229 −)

genes with fdr < 0.05 = 416 (195 + / 221 −)

genes with fdr < 0.01 = 370 (176 + / 194 −)

genes in genesets = 0

<FC> = 0

<t−score> = 0

<p−value> = 0

<fdr> = 0.77

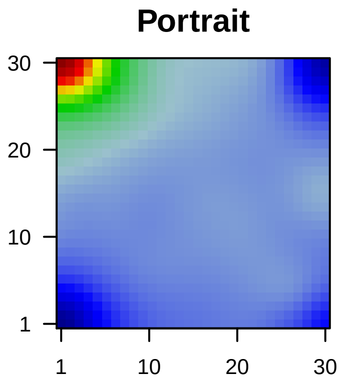

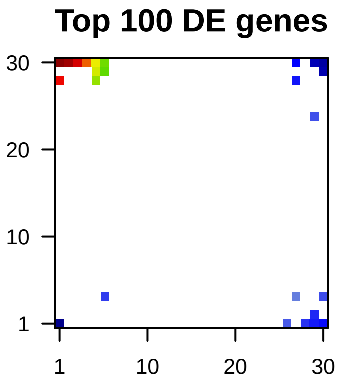

Global Genelist

Rank	ID	log(FC)	p−value	fdr	Metagene	Description
1	TRINITY_DN1	0.5	2e−16	2e−15	30 x 1	
2	TRINITY_DN2	−0.58	2e−16	2e−15	1 x 1	
3	TRINITY_DN9	−0.73	2e−16	2e−15	30 x 30	
4	TRINITY_DN7	−0.62	2e−16	2e−15	30 x 1	
5	TRINITY_DN7	−1.94	2e−16	2e−15	30 x 30	
6	TRINITY_DN5	−2.17	2e−16	2e−15	30 x 30	
7	TRINITY_DN5	0.94	2e−16	2e−15	1 x 30	
8	TRINITY_DN7	−1.19	2e−16	2e−15	1 x 1	
9	TRINITY_DN4	0.52	2e−16	2e−15	1 x 28	
10	TRINITY_DN4	2.03	2e−16	2e−15	1 x 1	
11	TRINITY_DN9	1.08	2e−16	2e−15	4 x 30	
12	TRINITY_DN9	−0.83	2e−16	2e−15	30 x 1	
13	TRINITY_DN9	−0.41	2e−16	2e−15	29 x 24	
14	TRINITY_DN9	0.49	2e−16	2e−15	4 x 30	
15	TRINITY_DN9	−0.47	2e−16	2e−15	30 x 30	
16	TRINITY_DN9	−0.84	2e−16	2e−15	30 x 30	
17	TRINITY_DN9	0.95	2e−16	2e−15	27 x 4	
18	TRINITY_DN9	0.58	2e−16	2e−15	6 x 4	
19	TRINITY_DN2	2.61	2e−16	2e−15	1 x 30	
20	TRINITY_DN2	−0.65	2e−16	2e−15	1 x 1	

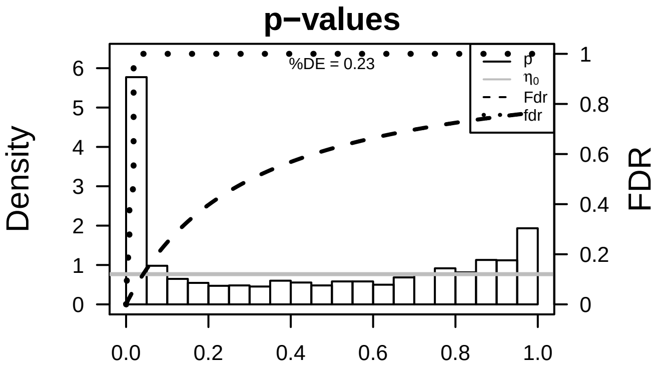

Supplement: Supplementary file 1 [file cells-09-00779-s001.zip › Supplementary materials/FileS4.zip!/FileS3/TFs - Results/Summary Sheets - Samples/Sprint.2.20.4..pdf]
